# Life is different now – impacts of eating disorders on Carers in New Zealand: a qualitative study

**DOI:** 10.1186/s40337-021-00447-z

**Published:** 2021-07-23

**Authors:** Louise Fletcher, Henrietta Trip, Rachel Lawson, Nicki Wilson, Jennifer Jordan

**Affiliations:** 1 Department of Medicine, University of Otago, Dunedin, New Zealand; 2 Centre for Postgraduate Nursing, University of Otago, Christchurch, New Zealand; 3 South Island Eating Disorders Service, Canterbury District Health Board, Christchurch, New Zealand; 4 Eating Disorders Association of New Zealand, Wellington, New Zealand; 5 Department of Psychological Medicine, University of Otago, Christchurch, Christchurch, New Zealand; 6 Specialist Mental Health Clinical Research Unit, Canterbury District Health Board, Christchurch, New Zealand

**Keywords:** Eating disorders, Carers, Burden of care, New Zealand, Thematic analysis, Qualitative

## Abstract

**Background:**

Eating disorders are widely recognized as serious illnesses, with complex psychological and physiological comorbidities. Affected individuals face a protracted and challenging treatment journey which, particularly for children and adolescents, requires significant input from family members as carers. This study investigated the perspectives and experiences of those caring for family members with an eating disorder diagnosis.

**Method:**

Participants were carers of affected individuals with an eating disorder, and were recruited from an online survey, subsequently consenting to a semi-structured qualitative interview. Inductive thematic analysis was undertaken to identify themes.

**Results:**

Most participants in the sample were parents of affected individuals. Significant and ongoing psychological and emotional impacts were identified across the sample. The diagnosis, treatment journey and overall impact of the carer role created a situation captured by the over-arching theme ‘life is different now’. Impacts profoundly influenced relationships and were felt across all aspects of life by carers, affected individuals and other family members. Heightened worry and vigilance experienced by carers continued beyond improvement or recovery.

**Conclusions:**

This research highlighted challenging and often exhausting impacts felt by carers due to their pivotal role in eating disorder treatment. Inadequate support for most carers in this sample has clear implications for families as well as service and funding providers. Further research should more fully investigate carer experience with different eating disorders to explore the type of support necessary to build capacity and resilience to reduce carer burden.

**Supplementary Information:**

The online version contains supplementary material available at 10.1186/s40337-021-00447-z.

## Plain English summary

Eating disorders (EDs) are serious illnesses with a challenging treatment journey that often requires significant input from family members as carers. This study investigated perspectives and experiences of those caring for family members with an ED diagnosis.

Carers reported significant impacts on many facets of their lives. The findings revealed levels of emotional and psychological stress both during and beyond the treatment journey. For carers ‘normal’ life was profoundly impacted by the complicated and often unanticipated role of treatment provider, and navigating the maze of treatment while trying to understand the diagnosis itself. For those with other children, the impacts were compounded by the distress also witnessed in those children. Relationships were significantly affected by the demands of treatment and the serious consequences of the disorder, as well as misunderstandings and stigma related to EDs.

A ‘new normal’ state of constant worry and ongoing vigilance defined life post-diagnosis. The results indicate the impacts of an ED diagnosis in a loved one, and the demands of caring for that person and assuming the role of treatment provider were life-changing. For these carers, ‘life is different now’.

**Supplementary Information:**

The online version contains supplementary material available at 10.1186/s40337-021-00447-z.

## Introduction

The significant physical risks and psychosocial impacts of eating disorders (EDs) are well-recognised [1–4]. Carers play a pivotal role in treatment, particularly for children and adolescents with an ED. The experience of being a carer of an individual affected by an ED (referred to as an affected individual (AI) has been associated with intense impacts pervading every aspect of their lives, documented in burden of care studies from Australia [5], the United Kingdom [4], and more recently the United States [3].

‘Burden of care’ is a complex construct encompassing objective and subjective emotional, physical, social, financial and psychological burdens which has been examined in the context of carer of people with a range of physical or mental illnesses [6, 7].

The complexity, unpredictability and seriousness of EDs challenge family dynamics, interpersonal relationships and social functioning. Symptoms of, and responses to EDs influence each other in a complicated, often drawn-out manner [8]. Furthermore, overcoming obstacles and barriers to access appropriate and timely care, and persuading a family member to participate in treatment can cause profound distress, fear and arduous burdens for carers, affected individuals (AIs) and family members [5, 9–11]. McDonald (2018) describes the sense of despair pervading the caring relationship when defined by fear (of the illness), and paralysis when carers do not know how to help [12].

Prevalence and patterns of EDs in New Zealand (NZ) at the time of our most comprehensive national epidemiology study [13] were largely consistent with international data at 1–4%, with higher rates in Māori and Pasifika populations. Adolescents in New Zealand are at particular risk for developing disordered eating attitudes and behaviours [13], consistent with international prevalence data [14]. ED treatment in NZ is informed by international research and treatment guidelines [15], and is based upon a multidisciplinary, patient-centred approach [15, 16]. Treatment options vary according to age, consumer need and service availability, mostly on an outpatient basis [15]. First line treatment for those under 18 is grounded in the Family Based Therapy (FBT) model developed in the 1980s at the Maudsley Hospital [17], subsequently manualised by Lock and Le Grange [18].

A NZ Ministry of Health (MOH) report issued in 2008 noted a lack of nationwide coordination of ED services making access to (and understanding of) treatment options difficult [19]. Subsequent MOH funding to address these issues included national rollout of FBT [20, 21]. Despite these improvements, the submission by the Eating Disorder Association of New Zealand (EDANZ) [22] to the Mental Health and Addiction Enquiry – He Ara Oranga (2018) [23] described continued gaps in treatment access, lack of service coordination, inadequate primary healthcare training, and a lack of accessibility to adequate care – consistent with problems reflected internationally [3–5, 24, 25]. International research also reveals significant financial, social and emotional costs to AIs and carers [3–5].

The treatment journey for those with an ED diagnosis, especially anorexia nervosa (AN), is very often protracted and complex, exacerbating the carer burden [3–5, 8] Carers play a pivotal role in treatment (especially if family-based treatment (FBT) is utilised) involving engagement with the multidisciplinary team. It may require repeated and or lengthy hospital admissions for the most physiologically compromised individuals [15]. Carers often report emotional burden related to lingering stigma and family blame for EDs. The carer burden in EDs is exacerbated by dealing with the illness itself, the challenges of ED treatment and recovery processes [26–28] and lingering stigma surrounding EDs, with families historically being ‘blamed’ [29].

Carers, their experiences and perspectives are an invaluable resource with the potential to inform policy and service provision development [23]. Although there is anecdotal evidence that NZ experiences for carers are likely to be similar to international reports, to the best of our knowledge, there are no published NZ data on costs, burden of care and treatment experience for NZ carers. This particular work is part of the Costs of Eating Disorders in NZ (the Costs Study) project, a mixed methods collaborative project [30].

## Method

### Research aim

This study aimed to investigate perspectives of those caring for a family member with a diagnosis of an ED, in terms of both their perception of treatment and the impacts of the ED on their experience of burden of care.

### Design

This study used a qualitative descriptive design and semi-structured interviews to focus on perspectives of treatment, impacts of the illness, and the burden of care, in order to provide an insight into the needs of ED carers, and the broader costs of EDs for this group. This methodological approach allows the data to speak for itself, provides tools for the researcher to review data within the context of participant experience and recognises the participant as an expert within the research phenomenon [31].

### Recruitment

Participants for this study emailed the researchers to volunteer for this study after completing the online survey for the Costs Study project [32] and were allocated a unique identifier used throughout this report. Recruitment for the survey was broad, including publicity through the EDANZ website, contacts within ED treatment provider networks and Māori health providers, a research group Facebook page, and hard copy posters disseminated across New Zealand in community settings.

Inclusion criteria were having been a carer for an AI with an ED (formally diagnosed or meeting criteria for a clinically significant ED) and to be able to read and converse in English. Carers were defined as parents, spouses or others who had taken on a significant support role, including siblings, friends or other relatives. There were no exclusion criteria.

Participants were provided with an information sheet and gave informed consent for the interview. The study had ethical approval (Northern B Health and Disability Ethics Committee, NZ 16/NTB/189/AM01). Interviews were conducted from December 2016 to December 2018.

### Data collection and management

Interviews were conducted by J Jordan (PhD, experienced clinical psychologist and qualitative researcher) and L Fletcher (Master of Nursing student, new researcher). Interview questions were devised by Jordan, Fletcher, and H Trip (PhD, registered nurse, qualitative researcher), in consultation with N Wilson from EDANZ (BA, carer, investigator) and R Lawson (Dip Clin Psyc, M.A., clinical psychologist, treatment provider, investigator). Eight interviews were conducted by phone and one was conducted face to face in a university research setting. They were typically around 40 min but ranged from 20 to 90 min. All participants were alone during the interview, although they could have had a support person if they wished. Interviews were conducted Both interviewers were female. Participants were aware of the researcher roles, that the team included clinician, service provider and carer perspectives, and of the purpose of the study.

Semi-structured interviews allowed participants to engage in questions relevant to the topic with room to talk freely [33, 34]. Each participant was interviewed once and was asked the following questions:

**Question 1:** “Please tell me about the biggest impacts of the eating disorder for you / your family?” If not mentioned, interviewers asked a prompt question about financial, psychological, social (family relationships, romantic relationships, friendships), education, work and other impacts.

**Question 2:** “Please tell me about the experience of treatment for you / your family member”. If not mentioned, interviewers asked a prompt question about difficulties and positive aspects.

Field notes were made at the time of the interviews. Interviews were audiotaped, transcribed, and stored in password protected electronic files on a secure server. Information that could identify the carer, the AI or the service provider was removed (JJ or LF), including the region where treatment took place. All interviews were checked to ensure accuracy of transcriptions. Participants were offered the opportunity to review and amend the transcript from their interview but none did.

### Data analysis

Inductive thematic analysis and coding methods initially delineated by Boyatzis and further defined by Braun and Clarke allowed coding to be undertaken after multiple readings of the raw data.^36, 37^ Categories drawn from this coding process were synthesised into themes. Initial coding was done by LF in consultation with supervisors (JJ and HT). Moderation and cross-checking of categories and codes was undertaken to ensure integrity of the data analysis. Data saturation was not utilised for this study primarily because we interviewed all participants who volunteered so there was no opportunity to get more participants using the same sampling method. Of interest though is that Braun and Clarke, experts in this method, have recently published a critique challenging the concept and importance of data saturation in thematic analysis, noting that decision making on when to stop analysis invariably has a subjective basis on researcher interpretation of their data, rather than being a concrete aspect of the dataset itself [35].

A COREQ checklist is attached as a supplementary document.

## Results

### The sample

Table 1 delineates sample demographics (*n* = 9) and diagnoses of AIs. Most participants were parents (*n* = 8, two fathers and six mothers) and one was a partner of an AI. Most (*n* = 6) were Caucasian females and the average age of participants was 51 years. The onset of disease in the majority (66%) of AIs occurred during adolescence. Only 16% of AIs were recovered at the time of interview.

**Table 1:** Information about carer participants (*n* = 9) and their AI’s eating disorder diagnoses

Participant	Gender	Relationship to AI	ED diagnosis of AI
C1	Female	Mother	AN
C3	Male	Father	AN
C4	Female	Mother	AN
C7	Female	Mother	AN
C12	Male	Father	BN (not formally diagnosed)
C13	Female	Mother	ARFID
C14	Male	Partner	BN
C15	Female	Mother	AN
C17	Female	Mother	BED

*Abbreviations*: *C* carer, *AI* affected individual, *ED* eating disorder, *AN* anorexia nervosa, *BN* bulimia nervosa, *ARFID* avoidant restrictive food intake disorder, *BED* binge eating disorder

#### Findings

The overarching theme emanating from the study data was **‘Life is different now’** - illustrating the core impact, fluctuating intensity and diversity of participant perceptions and experiences. Examples of this were articulated by four sub-themes: *Eating disorders are life changing*; *Treatment and therapy can work but…*; *Relationships are profoundly impacted by eating disorders*; and *The worry never stops*.

#### Sub-theme 1 – eating disorders are life changing

All participants reported the experience of having a family member diagnosed with an ED as life changing. The impact of caring for an AI reshaped carers’ priorities, affected life on multiple levels and exposed carers to situations and challenges for which they felt unprepared. Categories in this theme illustrated both negative and positive consequences of such life-altering scenarios, and also reflected lengths carers felt they would, or could, go to in order to aid recovery.

##### Serious and shocking

Carers reported an overwhelming sense of shock when their AI was diagnosed with an ED, exacerbated for the majority by the diagnosis being unanticipated. The shock, and subsequent realisation of the seriousness of the diagnosis, had profound psychological and emotional impacts: “*…the psychological impact in me personally… I have found it really difficult to cope … dealing with one of those unexpected life events that you never ever have planned for” (C17).* C1 reported that the diagnosis itself was in fact the biggest impact for her - “*… the worst impact was that she had it obviously.”*

The emotional and psychological strain accompanying the realities of an ED diagnosis, treatment and caring for an AI were expressed by participants as being more significant than the perceived financial impacts. C3 indicated that the financial impact “*didn’t end up being the priority*” and emotional strain *“[took] over your life”.*

##### Life is on hold

Carers stated that life, as it had been, went on hold. C4 stated that “…*the rest of my life kind of stopped*” as she focused on caring for her AI. This change in priority, while recognised by carers as essential, exacted a heavy toll on their own wellbeing and was “*… very, very draining*” (C13).

Treatment demands created significant burdens for carers, further compounded by emotional and psychological strain.

*We have no life, and I know I have had support counsellors try to tell me that ‘oh well you can’t let this rule your life, you need to get on with your life and live your life’. Well that is actually not possible when you are still having to supervise every meal and snack every day and be so vigilant. (C15)*

##### Doing what needs to be done

Despite the significant impacts experienced by carers, all expressed being highly motivated to do whatever was required in order to help their AI through the treatment journey. C1 stated “*…because you just want to do the best right, you just want your kid well and you don’t care. I mean obviously within a limit but … you just want the best care for your kid.”*

C4 asserted that there was simply no question, the only choice she had was to fight the illness for her child. “*They would go, ‘well what are you going to do if [AI] dies?’ and I said ‘he’s not going to die because I have a choice, I can stop this.”* Carers often gave up work, study and personal pursuits in order to care for their AI, expressing that they felt lucky to be able to do so. When talking about the demands of refeeding her child during inpatient care on a general hospital ward, C1 stated:

*I just gave up my job and I did it, but there were other families who couldn’t do that and those kids … sometimes they just wouldn’t have their lunch … because you had to have somebody to sit with them and there wasn’t somebody available.*

##### Silver linings

Despite the trauma and significant impacts of caring for an AI, participants generally expressed a sense of being able to find positives in their situation. When talking about her AI’s challenges in dealing with change C1 stated:

*I mean obviously I wish she hadn’t had to go through this experience to learn it but I actually think that is one of the best outcomes for her, because I think that as she goes through her life she will be able to cope with these things better than had she not had it.*

Carers also spoke about changes in priorities and shifts in focus having a positive spin-off on family dynamics. C13 reflected that *“… it actually made us spend family time and I think it’s probably bought us together as a unit more”.* Carers also recognised ways in which sibling relationships were reinforced by the shared experience. Reflecting on a long treatment journey C12 stated that his children had grown closer because “*…[AI] has confided in [sibling] … he is very understanding and supportive … he realised that it is very important to continue to support her as she goes through this*”.

Carers also expressed gratitude for this shared experience increasing understanding between parent and child. “*There is that shared experience … we have sort of been through a fair bit together … there is a little bit of mutual respect that is built up out of that*” (C3).

#### Sub-theme 2 – treatment and therapy can work ‘but’

The majority (66%) of AIs in this sample underwent a combination of inpatient hospital care and FBT for anorexia nervosa (AN). The remainder of the sample utilised various therapies including Cognitive Behavioural Therapy (CBT), Dialectical Behaviour Therapy (DBT), psychotherapy and counselling. Participants described the treatment journey as challenging for them, their AI and for the wider family, and this sub-theme identified that treatment was a necessary but difficult process.

##### A significant burden of responsibility

Carers reported an overwhelming sense of responsibility for providing treatment, with this burden increasing the already significant emotional and psychological impacts of the illness.

*I wonder if she had cancer whether I would have had that same amount of responsibility put on me, because I feel like she would be given an oncologist who would take responsibility for her, for her type of cancer, yet in this illness that is a massive responsibility on a parent who is not trained to take that on. (C15)*

Some carers expressed the view that this burden of responsibility was exacerbated by a sense that they were failing in their fundamental ability to care for their child. C13 stated this was “*confronting*” and that she found herself asking “*what have we done wrong and what are we still doing wrong*”. Similarly, carers felt the demands of prolonged, unpredictable treatment had the tendency to outshine perceived successes along the way.

*When it had gone well and she had put on a kg … we’d be like ‘Woohoo! We’ve done our job!’ … and then we’d get into the therapy and we’d start feeling ‘Bleurgh – right, still got work to do’. (C13)*

The majority of carers expressed the view that having to coordinate the various, often complicated aspects of treatment programmes increased this burden of responsibility.

*I have had to be the project manager of her treatment and that has felt like a really huge responsibility on me because I am not medically trained at all and I have to take on board being her total advocate, her project manager for her health care. (C15)*

##### Navigating the maze

Carers expressed a sense of having to navigate their way through the system in order to access treatment, adding to the stress of the situation.

*The biggest sort of frustration for me is really … and you know I … know how to get through the maze of information and bureaucracy … but somehow with this, I just couldn’t find, haven’t really still now, been able to find a way through the maze. (C17)*

C1 reflected on her sense of frustration at having to ensure appropriate follow-up care was put in place, stating that *“…we managed to get a psychologist, but only because I kicked up bobsy-die* [making a fuss] *and you shouldn’t have to do that*”. When coordination of care was effective carers reflected this helped in their efforts to care for AIs. C17 noted that treatment would be significantly less stressful if it was “*…all under one umbrella*”.

##### Waiting makes it worse

While trying to access or commence treatment, carers reported symptoms of the illness often escalated with a resultant deterioration in the condition of their AI.

*I took her first to a psychologist that we knew and we had a couple of sessions with her and then even in that, I think we had to wait a couple of weeks for an appointment, and then a week between appointments, in that month she deteriorated really quickly. (C15)*

The seriousness of an ED, and the often-rapid progression in physiological consequences in AIs, meant that for many carers, the delay in accessing treatment added to their distress. This continued throughout the treatment process with C4 reflecting that “*everything just takes, you know, two days too long”.*

##### The importance of communication and information

Carers reported that access to reliable information about the diagnosis and treatment plan, and effective communication between those involved in the care of their family member, in part mitigated some stressors. “*The nursing staff were really good … [it] was really valuable to get feedback from them and give them feedback too about what I was seeing. (C15).*

Conversely when communication across members of the treatment team was not effective, it appeared to add to the overall burden as carers felt stressed when they had to chase and then decipher different information from different sources. They had to step up to take on the role of facilitating communication between service providers where this inter-agency communication was insufficient. “*That was sometimes a struggle when you’ve got crossover between mental health services and services that are more geared towards an ED, there didn’t seem to be a good deal of communication between the two*” (C3).

The importance of effective communication and appropriate information was particularly highlighted by those supporting an AI in treatment for AN. The initial focus on refeeding in FBT appeared to be confusing as it seemed to ignore the anxieties and psychological symptoms of the AI. One mother expressed the distress she felt when she believed treatment was ignoring the general anxiety she was seeing in her AI:

*It was just about getting her to eat and then someone just said ‘well now, they’re not functioning, their brains aren’t working’ … and I’m like ‘oh why didn’t you just…*[tell us that]*… cos I thought you guys were ignoring half the problem, most of the problem. (C13)*

Once the rationale behind refeeding was explained, carers once again appeared to embrace the notion of doing ‘whatever it took’ to help their family member recover. “*So that … was my single goal with it. It was like nothing else actually matters*” (C4).

Throughout the interviews, carers provided insightful, considered opinions on what might make treatment experiences easier, or more effective. The need for reliable and accessible information emerged clearly from the data, as did the need to avoid minimizing the condition or generalizing about the potential course of the disease and treatment journey. “*Maybe more information upfront … because D, [it] was always like ‘oh she’s young’, ‘ARFID’s ARFID’, ‘she should be in and out quicker’, and that’s not necessarily the case*” (C13).

##### Challenges and successes

Despite their expressed frustrations, many carers believed that FBT helped and was an essential part of the recovery of their AI.

*It was positive because she was critically unwell and it got her better right? I mean we probably could have done it at home but it probably would have been harder, so I think that experience, even though I have talked to you about the negative things, overall that was positive. (C1)*

The sense of treatment providing a framework for recovery was also evident in the data, particularly when carers were adjusting to the reality of the diagnosis, treatment and potential prognosis. C3 stated that treatment gave him “*… some stock to hang things on*” and “… *some regularity*” at a time of significant uncertainty.

Some carers also found being taught externalisation techniques, where they were encouraged to separate the disorder from their AI, enabled them to remain strong, with one mother identifying the disease as a Harry Potter dementor “…*and we [were] fighting that dementor as a family and so the label was quite useful*” (C13). This ability to externalise the disease assisted carers in coping with challenging behaviour resulting from the illness. C4 stated that without the externalisation technique “*… it would completely kind of damage your relationship*”.

##### Battling the system

Many carers expressed frustration at apparent incoordination of services, seemingly inadequate knowledge of some medical staff about the complexities of ED treatment, and exacerbation of carer burden in facilities and environments which appeared to be inappropriate and an impediment to recovery. The unsuitability of a medical ward for ED treatment translated into heightened stress for both carers and AIs, with C7 stating that she did not feel that there was “*…anybody in that hospital that understands EDs*”. Carers also expressed frustration and anger at attitudes of medical staff who seemed to lack training in how to deal with those with an ED diagnosis.

*So when we were admitted one of the nurses came in and said ‘right young lady we are going to sort you out’, you know like she was 15, and you know this kid was 10 and it is like, just totally not ok. (C1).*

#### Sub-theme 3 – relationships are profoundly impacted

Impacts of EDs on relationships were often distressing, and carers reported significant impacts on others within the family, particularly siblings.

##### Changing roles – parent and child

Carers reported their previous parental role was suddenly changed by the demands of ED symptoms and treatment. Resultant distress was summed up by C17 who stated that she “*…didn’t know I guess what to do or how to help or what our role was*”.

There was a sense of the ED fragmenting family unity as parents navigated new roles in order to help their child in the various stages of their illness. “*My husband was very good at talking to her, and reasoning with her and I was the one who just sat with her for hours while she ate* (C7).

During inpatient refeeding, particularly in general medical wards, carers found themselves in the role of treatment provider for which many felt unprepared, rather than in their primary role as parent to their child. “*So I was basically the main caregiver, and I mean the nurses were very grateful but… that is all fine but it is not really appropriate either*” (C1). In spite of the exhaustion and frustration she expressed about the demands of treatment, C15 recognised the necessity of this change in her role, stating that “*she feels safer if I am there telling her to eat it, if I am not there telling her to eat it, then the ED will tell her not to*”.

##### EDs split families

For some carers the need to stay with their AI during inpatient treatment necessitated long periods away from other family members, including other children. Expressed by C1 as being “… *basically split in two*” by competing demands of treatment and family, this impact continued during outpatient FBT. Carers expressed feeling torn between having to provide intensive treatment and focus for their AI, while simultaneously trying to support other children. “*Obviously we spent a lot of time explaining to [sibling] but at the same time we are desperately trying to get this other kid to eat you know*” (C1).

##### Siblings are hurt too

The notion of a family being split by the disorder was particularly evident in data from families where the AI was also a sibling.

*So for my daughter she has lost her sister. … We get a few windows here and there but it has affected the relationship, so I guess [sibling] is on edge when she’s around [AI] and maybe quite defensive, you know, it has affected the relationship. (C15)*

The time and energy required for carers to adequately care for their AI translated into a sense of having neglected other children, where treatment challenges and the illness itself “… *certainly impacted on them and sucked up time*” (C13). For some this developed into resentment among siblings, which appeared to be exacerbated by expectations that siblings were also responsible for helping their AI through treatment. C13 elucidated this when she said “*… there is probably some resentment and now we’re back to her sitting at the table, making sure she’s eating her dinner, and, everybody’s on her you know*”.

For those carers undergoing FBT, there was a sense that, although ‘family based’ there was not enough support within the treatment programme for others in the family. In particular, carers felt effects of the illness on siblings needed more attention and support with C1 noting that for her daughter “... *it was very difficult for her to see her sister like this, and … try and understand what is going on”.*

Carers often reported siblings found therapy sessions, particularly FBT, challenging and overwhelming. “*…the middle one would shut down and wouldn’t say anything … I’d ask her a question she’d say, ‘I don’t know’ … that’s just her coping mechanism*” (C13). This strain on the sibling relationship appeared to be alleviated by a gap in ages between siblings. One mother reported that her older daughter, who had left the family home at the start of the ED journey, was only minimally affected by the realities of, the situation “*... cos she wasn’t there to see it*” (C7). Similarly, a mother whose other children were all younger than their AI reported that impacts of the illness were mitigated somewhat. “*They only really noticed a change of attitude. … They saw a nicer person come out the other end*” (C4). However one carer with both a relatively young AI and a younger second child reported that the trauma of the illness had a significant impact on her youngest child. C1 stated “…*that is pretty traumatic and … to see your sister in that kind of state is not very good*”.

##### Strained partnerships

One of the participants in this project was a spouse/partner to the AI. When talking about the impacts of caring for his partner, he indicated activities which would normally be a mainstay in a partnership were no longer possible due to the psychosocial challenges his partner faced as a result of her illness. This significantly altered their relationship dynamics.

*I’ve been involved with my partner for [several years] and her ED has sort of got worse and worse, and initially I didn’t really … know the extent of it, and now I’ve … assumed … caring for her.* (C14)

Other carers reported the potential strain on spousal relationships and the importance of having a strong and supportive partnership.

*It’s all tensions you know, and we had tensions that I was doing everything and he wasn’t and he could hide away at work … if you’ve got issues in your marriage already it would also make it even harder … there’s this huge pressure.* (C4)

Carers recognised the stress caused by the situation and the extraordinary demands placed on their partners as well as themselves.

*This was all going on all the time and I would end up ringing my husband in tears … because I was really worried about her and he’s trying … to look after [sibling] and have a job … it was pretty full on.* (C1)

Carers also expressed an appreciation for the way in which the demands placed on both them and their spouse strengthened their relationship. “*Certainly my husband I are a very tight unit, we always were but … we’ve had to be, in other ways now*” (C13).

##### Social relationships – a lonely journey

Participants expressed the loneliness felt as they navigated the demands of treatment, often at the expense of normal social interactions, combined with perceived stigma. C17 indicated that she initially “… *didn’t wanna tell anybody, and over time I’ve sort of managed to tell a few people*”. This sense of isolation appeared compounded by the weight of responsibility carers felt for providing care and treatment for their AI. “*It’s just, you live and breathe it and other people can’t do much*” (C17).

A sense of stigma and concurrent misunderstanding significantly impacted interpersonal relationships. “*Some friends still don’t know … some who are quite good friends … you just get a sense that some people will kind of understand more than others*” (C7). One carer even expressed the opinion that if her AI had been diagnosed with a medical illness, the support and understanding from those around her would have been vastly different. “*Because there are times where I have actually wished that she had cancer, not just for the medical care difference but also how the community would respond*” (C15). Stigma and shame expressed by the AI also limited the ability of carers to enjoy social interactions in a way they used to. “*We had to be very picky about when we had friends around for dinner … because initially she didn’t want anybody to know*” (C7).

##### EDs change relationships of AIs

Carers also saw a change in the dynamics of relationships their AI had with others as a result of the illness and treatment impacts. Although carers did report some silver linings in terms of improved relationships, most carers described negative impacts. “*She wouldn’t see anybody when she was in hospital … she really disconnected with all her friends*” (C1). One carer noted the significant impact the ED had on his AI’s ability to interact socially because it “…*knocked his confidence in dealing with other people*” (C3).

Carers reported AIs appeared to want to separate the ED from their normal life, with this continuing post-recovery. “*Even now … I said ‘well you know you could tell your friends’ and he doesn’t want to, he really struggles to go back there, he just struggles with the whole thing*” (C4).

#### Sub-theme 4– the worry never stops

For carers, the worry and resultant vigilance accompanying an ED diagnosis never ceased, even when they felt their AI was significantly recovered. There was now a pervading sense of this change to an active ever-vigilant parental role being their ‘new normal’ - that they could not relax back to usual parenting behaviours for offspring at that developmental stage.

##### Constant worry and vigilance

As carers progressed through treatment with their AI, many reported finding it difficult to move on from constantly worrying about the potential for recurrence and relapse. “*You know it is still hard not to worry … my husband and I both worry about [AI] anyways, even though we know she is well, we know how quickly things can go downhill*” (C1).

For others, progress was overshadowed by worry over what might happen when family circumstances changed. C13 was concerned about her impending return to fulltime work and *“… what happens if we strike issues again*” particularly given the demands and time commitment required during therapy and treatment. Carers also reported a heightened sense of vigilance now being a daily part of their lives. “*You wonder whether you can feel you can ever turn your back again because you think it was always going to happen again*” (C3).

Carers also reported concern about the potential for an ED to affect other children in the family. “*Obviously I am acutely aware of things because I’ve got a younger daughter. My level of paranoia is probably through the roof!*” (C1). Even when carers felt their AI was recovered there were subtle changes evident in daily routines reflecting their sense of vigilance, such as when going out to dinner C7 and her husband would “… *make sure she had her dinner first*”. For others whose AI was still struggling significantly with the effects of the illness, this vigilance translated into an inability to lead a normal life or undertake normal activities. “*The constant vigilance required 24/7, I can’t get a babysitter in for my 16-year old … who do you get to babysit when she might kick off?*” (C15).

##### The new normal

Carers reflected that caring for an AI with an ED meant that life was irrevocably changed and impacts of the ED were “… *always gonna be there, just in the background*” (C7).

In a similar way, carers noted their expectations of life for them and for their family member, were often quite different than they were before the diagnosis. “*You are quite happy if they generally seem in a good mood and are eating ok, that is a bonus, you are grateful for some of the smaller things*” (C3).

## Discussion

This study identified that impacts of an ED diagnosis in a family member are life-changing for carers in this NZ sample. The shock of diagnosis, coming to terms with the realities of the serious consequences of the illness, and the challenges of understanding a complicated treatment journey all weighed heavily on carers. Their lives post an ED diagnosis were significantly reshaped by the challenges and demands of treatment for the ED, and the journey towards recovery.

The present results are consistent with a comparative qualitative study undertaken in Australia identifying challenges for carers in accessing and navigating treatment, and revealing heavy demands placed upon carers, while simultaneously illustrating the strength and resilience of those in the carer role [9]. The positive findings of increased resilience and a recognition of strength and familial bonds are concepts that would benefit from further exploration. In particular, it may be useful to explore the concept of posttraumatic growth in carers – this has been noted in other contexts where a traumatic situation requires people to find or learn new adaptive skills to deal with the extraordinary demands placed upon them. This concept has been under-researched in carers of those eating disorders, despite the likely trauma experienced by carers witnessing a life-threatening condition such anorexia nervosa in their AI.

The results also align with reports from Australia, the United Kingdom and the United States which revealed significant social, economic and personal impacts on both AIs and those caring for them [3–5]. The current study contributes important insights into impacts of EDs on carers in the NZ context, consistent with international studies that highlight an often-hidden population of carers of those with eating disorders, with stigma and shame about the disease still felt across the sample. A number of international studies have noted that the far-reaching impacts on those caring for a family member with an ED diagnosis, raising the question as to whether these conditions ought to be considered as chronic and long-term, in order to provide adequate and relevant support to carers [3–5, 8, 9, 27, 36].

Difficulties in accessing, navigating and understanding treatment compounded burdens for participants in this study. Inappropriate facilities in some regions, lack of understanding about the needs of AIs and ineffective communication across treatment teams increased the frustration and isolation felt by carers. These findings are echoed in those reported by EDANZ [22], reflecting the complicated and sometimes fragmented system of care described across mental and general health services in NZ [37].

The present study found treatment often relied on significant effort and input from carers which is consistent with international literature [8, 11, 12]. Participants involved in FBT in this study reported a significant burden of responsibility was placed on family members without commensurate provision of support, a problem also noted internationally. ^(e.g. see^ [36]^)^ Carers reported this burden was keenly felt by siblings. A challenge for service providers is that the name of the treatment – ‘family based’– has the potential to be misconstrued by family members as providing input for the entire family, when in fact the focus of FBT is treatment for the AI. Concurrent roles as a parent or partner were profoundly impacted as carers took on a pivotal, complicated and time-consuming role as treatment provider. This shift in relationship dynamics was also observed in AIs, and the significant impact on siblings has been reported previously [10].

Carer burden did not dissipate when their AI entered the recovery phase. Constant worry and vigilance accompanying the experience of caring for their AI was ubiquitous and continued to influence daily life for carers. Although it is likely that some AIs with early access to treatment respond quickly, the findings from this current study align with international findings indicating that if illness persists, the burden of care becomes correspondingly heavier, with carers suffering from lack of respite and support, as observed in other chronic health conditions [12, 36].

### Limitations and strengths

There are some limitations to the study. Participants in this sample were all carers who have been profoundly affected by EDs, with most still involved in treatment and care for their AI with resultant heavy demands on time and coping resources. As such, this self-selected sample may not be representative of the wider population of ED carers. Although this study included some ethnic and gender diversity, most participants were Caucasian females and mothers. Future research would benefit from both targeted and random sampling of the ED carer population to enhance generalisability of the findings across eating disorders and include more diversity in age groups, gender and ethnic subgroups. Researcher perspectives were made transparent and the potential for researcher bias was discussed within the data analytic team on a number of occasions. The inclusion of researcher, clinician /service provider and carer/support organisation perspectives within the research team was helpful in minimising risk of bias.

### Recommendations

The findings of this study have the potential to inform development and funding of ED treatment and service delivery in New Zealand by providing new information about burden and unmet needs for New Zealand carers. The findings provide support for carer advocacy perspectives in submission processes for both the updated Carers Strategy [38] and the Health and Disability System Review [37].

Carers’ needs for adequate explanation of treatment processes should be communicated to treatment teams and providers and that information needs to be made easily accessible in a coordinated and consistent manner between AI, carer and treatment teams. More information about management of EDs needs to be disseminated across primary, secondary and tertiary healthcare providers.

Collaborative care training [11] developed by Professor Janet Treasure in the United Kingdom which aims to improve carers’ well-being, problem-solving and coping skills, has been successful in increasing the resilience of carers [26]. It is available in some ED treatment services in New Zealand, however there may be scope to further develop this training among FBT therapists in New Zealand. A national review is recommended regarding what support is available to families across services and regions and how that support is communicated to, and accessed by, those in need. Support from organisations such as EDANZ is already available and valued by carers. It may be that more targeted support (including peer-delivered support or mentoring) for siblings and other family members could be made available. In particular, findings from this study regarding carers’ concern about siblings and other family members should be considered by ED treatment providers. For example, there may be capacity to include an option for additional targeted support for siblings and other family members in referral pathways. In addition, treatment approaches such as Adolescent and Parent Treatment [39] that offer separate treatment and support to parents and AIs (without involving siblings) have the potential to reduce sibling reported where they have felt obliged to be involved in FBT.

## Conclusions

The role of carer is an essential component of ED treatment. Findings from this qualitative NZ study confirm that NZ carers report similar experiences of burden of care to that reported in international studies. This study also highlights important aspects of the carer role including its considerable burdens, yet simultaneously, its capacity for positive outcomes. These carers reported however that support was often insufficient to foster the resilience and sustainability required to enable them to continue in this provision of care.

## Supplementary Information

**Additional file 1.** COREQ (COnsolidated criteria for REporting Qualitative research) Checklist.

## Data Availability

The qualitative datasets generated and analysed during the current study are potentially available for ethically approved research from the corresponding author on reasonable request.
